# Nitric Oxide Enhances Rice Resistance to *Rice Black-Streaked Dwarf Virus* Infection

**DOI:** 10.1186/s12284-020-00382-8

**Published:** 2020-04-14

**Authors:** Rongfei Lu, Zhiyang Liu, Yudong Shao, Jiuchang Su, Xuejuan Li, Feng Sun, Yihua Zhang, Shuo Li, Yali Zhang, Jin Cui, Yijun Zhou, Wenbiao Shen, Tong Zhou

**Affiliations:** 1 Key Laboratory of Food Quality and Safety, Institute of Plant Protection, Jiangsu Academy of Agricultural Sciences, Nanjing, 210014 Jiangsu Province China; 2 College of Life Sciences, Laboratory Center of Life Sciences, Nanjing Agricultural University, Nanjing, 210095 China; 3 School of the Environment and Safety Engineering, Jiangsu University, Zhenjiang, 212013 Jiangsu Province China; 4 College of Resources and Environmental Sciences, Nanjing Agricultural University, Nanjing, 210095 China

**Keywords:** Rice, *Rice black-streaked dwarf virus*, Nitric oxide, Sodium nitroprusside, *Osnia2* mutant rice

## Abstract

**Background:**

*Rice black-streaked dwarf virus* (RBSDV) causes one of the most important rice virus diseases of plants in East Asia. However, molecular mechanism(s)controlling rice resistance to infection is largely unknown.

**Results:**

In this paper, we showed that RBSDV infection in rice significantly induced nitric oxide (NO) production. This finding was further validated through a genetic approach using a RBSDV susceptible (Nipponbare) and a RBSDV resistant (15HPO187) cultivar. The production of endogenous NO was muchhigher in the 15HPO187 plants, leading to a much lower RBSDV disease incidence. Pharmacological studies showed that the applications of NO-releasingcompounds (i.e., sodium nitroprusside [SNP] and nitrosoglutathione [GSNO]) to rice plants reduced RBSDV disease incidence. After RBSDV infection, the levels of *OsICS1*, *OsPR1b* and *OsWRKY 45* transcripts were significantly up-regulated by NO in Nipponbare. The increased salicylic acid contents were also observed. After the SNP treatment, protein S-nitrosylation in rice plants was also increased, suggesting that the NO-triggered resistance to RBSDV infection was partially mediated at the post-translational level. Although *Osnia2* mutant rice produced less endogenous NO after RBSDV inoculation and showed a higher RBSDV disease incidence, its RBSDV susceptibility could be reduced by SNP treatment.

**Conclusions:**

Collectively, our genetic and molecular evidence revealed that endogenous NO was a vital signal responsible for rice resistance to RBSDV infection.

## Background

*Rice black-streaked dwarf virus* (RBSDV) is a member in the Genus *Fijiviru*s, family *Reoviridae*. RBSDV is known to be transmitted by small brown planthopper (SBPH, *Laodelphax striatellus*) in a persistent manner (Shikata and Kitagawa 1977; Hibino 1996; Feng et al. 2019), and can cause severe damages to rice (*Oryza sativa*), maize (*Zea mays*), and several other cereal crops in the eastern region of Asia. RBSDV symptoms in rice plants include plant stunting, leaf dark greening, pale green enation formation, and dark color leaf vein swellings (Bai et al. 2002; Chen and Zhang 2005; Lee et al. 2005). It was previously reported that phytohormones played crucial roles in rice resistance to RBSDV infection. For example, during RBSDV infection in rice, jasmonic acid (JA) production was found to be increased; whereas, brassinosteroid (BR) production, which is important for integrating salicylic acid (SA) pathway with jasmonic acid (JA) pathway, was suppressed (He et al. 2017; Pan et al. 2018). A recent study further showed that abscisic acid (ABA) could negatively regulate rice resistance to RBSDV infection (Xie et al., 2018).

In addition to above phytohormones, nitric oxide (NO) can also modulate plant and animal defenses against biotic and abiotic stresses (Schmidt and Walter 1994; Delledonne et al. 1998; Mur et al. 2006; Gaupels et al. 2011). NO can be produced through the nitrate/nitrite-dependent pathway that is known to be catalyzed by nitrate reductase (NR) (Gupta et al. 2011). In Arabidopsis, *nitrate reductase1 and 2* (*AtNIA1/NIA2*) genes were reported to be involved in the NR-dependent pathway (Lozano-Juste and León 2009; Lozano-Juste and León 2010). An earlier study showed that ABA could enhance NO production in plant guard cells to regulate stomatal closure (Neill et al. 2002). *AtNIA1* and *AtNIA2* mutants showed impaired stomatal closure due mainly to the altered expressions of core genes involved in ABA signaling, and the impaired stomatal closure could be restored by the applications of exogenous NO (Zhao et al. 2016). In addition, NO can be produced through _L_-arginine-dependent pathway that is known to be catalyzed by mammalian NO synthase (NOS)-like enzyme (Crawford 2006; Besson-Bard et al. 2008; Simontacchi et al. 2015). In Arabidopsis, *nitric oxide-associated1* (*AtNOA1*) gene was shown to be related to the _L_-arginine- or NOS-dependent pathway (Sanz et al. 2014). NOS-dependent NO production was increased during the host response to *Rhodococcus* and *Streptomyces*, demonstrating the induction by host signals (Cohen and Yamasaki 2003; Johnson et al. 2008). It was reported that NO could regulate the interactions between plants and pathogens (Hong et al. 2008; Arasimowicz-Jelonek and Floryszak-Wieczorek 2014). For example, NO produced in tobacco could induce cell death to defend *Pseudomonas syringae* Pathovars (Mur et al. 2005). NO is also important for Arabidopsis resistance to *Sclerotinia sclerotiorum* (Perchepied et al. 2010). It was previously reported that NO could be produced during the interaction between plant and pathogen through the phytohormone-dependent signaling. Song and Goodman (2001) discovered that NO could regulate the SA-induced plant resistance against *Tobacco mosaic virus* (TMV) infection. To date, the function of NO in virus infection, especially in rice, remains largely unknown.

*S*-nitrosylation-mediated protein post-translational modification has been used to investigate the physiological functions of NO during animal and plant stress responses (Jaffrey and Snyder 2001; Lindermayr et al. 2005; Yun et al. 2011). Feechan et al. (2005) showed that the increase of AtGSNOR1 (*S*-nitrosoglutathione reductase) activity could induce wheat resistance against wheat powdery mildew invasion. *S-(hydroxymethyl)-glutathione dehydrogenase* deletion mutants in *Magnaporthe oryzae* were sensitive to the NO application, and could produce more *S*-nitrosothiols (SNOs) than the wild type *M. oryzae* (Zhang et al. 2015). However, this NO-dependent *S*-nitrosylation has not been shown to be involved in rice resistance to virus infection.

 Rice cv. Nipponbare is known to be susceptible to RBSDV infection (Lan et al. 2018), and rice cv. 15HPO187 was found to be resistant to RBSDV infection in our earlier field surveys. In this study, these two cultivars were used to study the role(s) of NO in rice resistance to RBSDV infection. We first compared the levels of endogenous NO in these two rice cultivars during RBSDV infection. The effects of NO-releasing reagents or NO scavengers on RBSDV infection in these two cultivars, were assessed. The possible cross-talks between NO and SA, and the NO-dependent *S*-nitrosylation were subsequently investigated. Because the endogenous NO level was reported to be suppressed in Arabidopsis and rice *nia2* mutant plants (Wilkinson and Crawford 1991; Fan et al. 2007; Cao et al. 2008; Sun et al. 2016), the rice *Osnia2* mutant plants were used to validate our pharmacological results on the function of NO in rice resistance to RBSDV infection. Our genetic results further indicated that NO might be a key regulator of rice resistance to RBSDV infection, at least partially, through a salicylic acid-dependent signaling.

## Results

### 15HPO187 Plants Were Resistance to RBSDV Infection

**Fig. 1:**
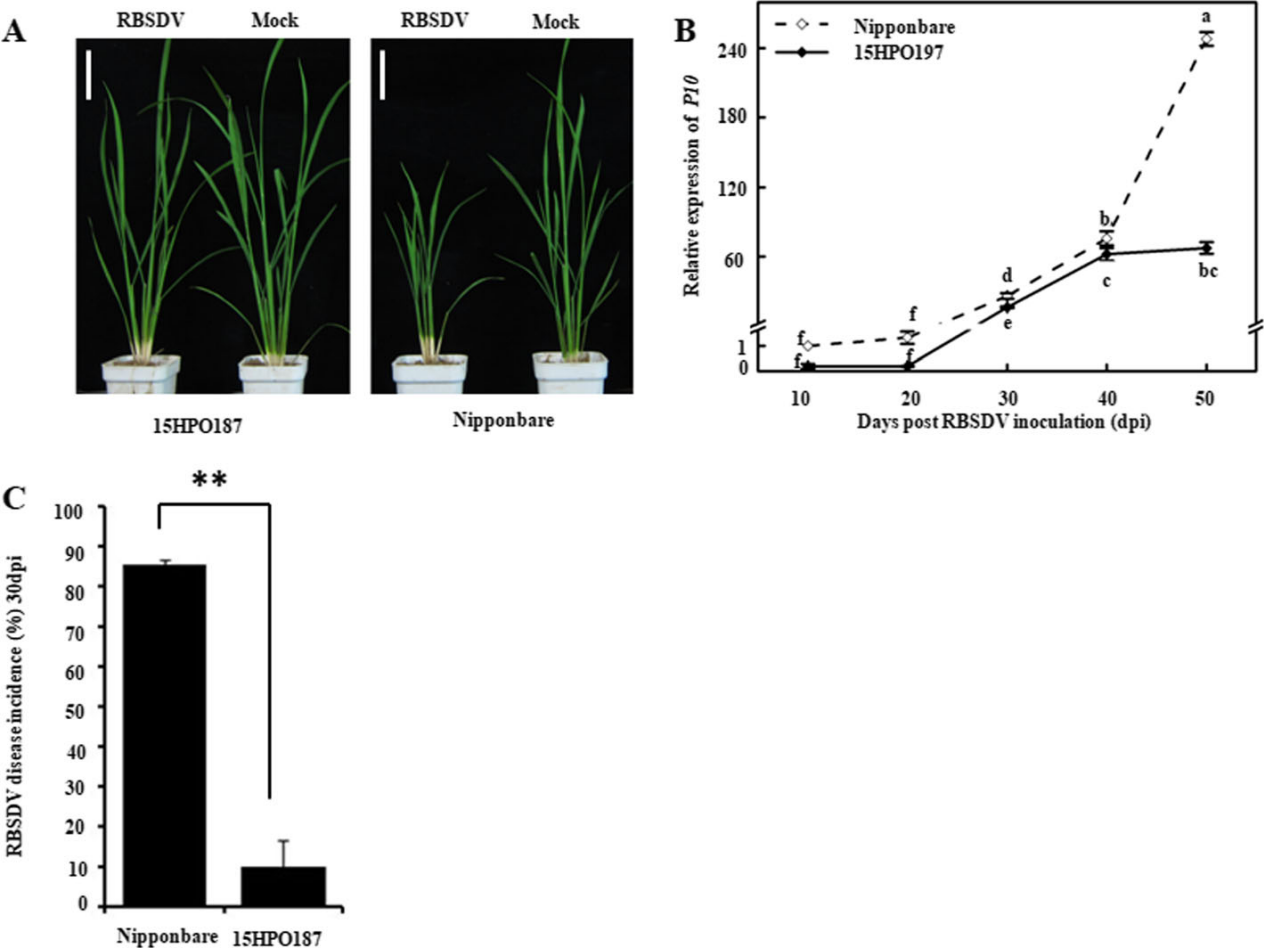
The plant growth of Nipponbare and 15HPO187 challenged with RBSDV infection. **a** A mock- or RBSDV-inoculated Nipponbare (left) or 15HPO187 (right) plants photographed at 30 dpi. **b** Time-course analysis of RBSDV *P10* gene in the RBSDV-inoculated Nipponbare or 15HPO187 plants at various dpi. Five plants were pooled together as one biological experiment for each time point for analysis. The data represented the means ± SD of three biological replicates. Entire experiment repeated 3 times with similar results. **c** RBSDV disease incidence of the Nipponbare and 15HPO187 rice was determined at 30 dpi. 30 two-leaf stage rice seedlings of each cultivar were tests for disease incidence experiment. Scale bars = 5 cm. Images are representative of three independent biological experiments. The data represented the means ± SD of the three replicates. Different low case letters above the bars indicate the statistical differences between the treatments using the Duncan’s multiple test, **, *p* < 0.01. Mock, plants were inoculated with non-viruliferous SBPHs; RBSDV, plants were inoculated with RBSDV viruliferous SBPHs

Nipponbare and 15HPO187 seedlings were inoculated with RBSDV viruliferous or non-viruliferous SBPHs. By 30 dpi, the RBSDV viruliferous SBPH-inoculated 15HPO187 plants showed mild leaf darkening and twisting symptoms; while, the RBSDV viruliferous SBPH-inoculated Nipponbare plants showed strong leaf darkening and twisting, and plant stunting (Figs. 1a, S1). Quantitative RT-PCR using RBSDV *P10* ORF specific primers showed that RBSDV RNA accumulated similar in both RBSDV-inoculated 15HPO187 and Nipponbare plants at 10 and 20 dpi, but RBSDV RNA accumulated in 15HPO187 plants was much lower than in Nipponbare plants at 30 dpi (Fig. 1b). Consistently, approximately 85% of the RBSDV-inoculated Nipponbare plants showed virus symptoms, while only about 10% of the RBSDV-inoculated 15HPO187 plants showed virus symptoms (Fig. 1c).

### RBSDV Infection Altered Endogenous NO Metabolism

**Fig. 2:**
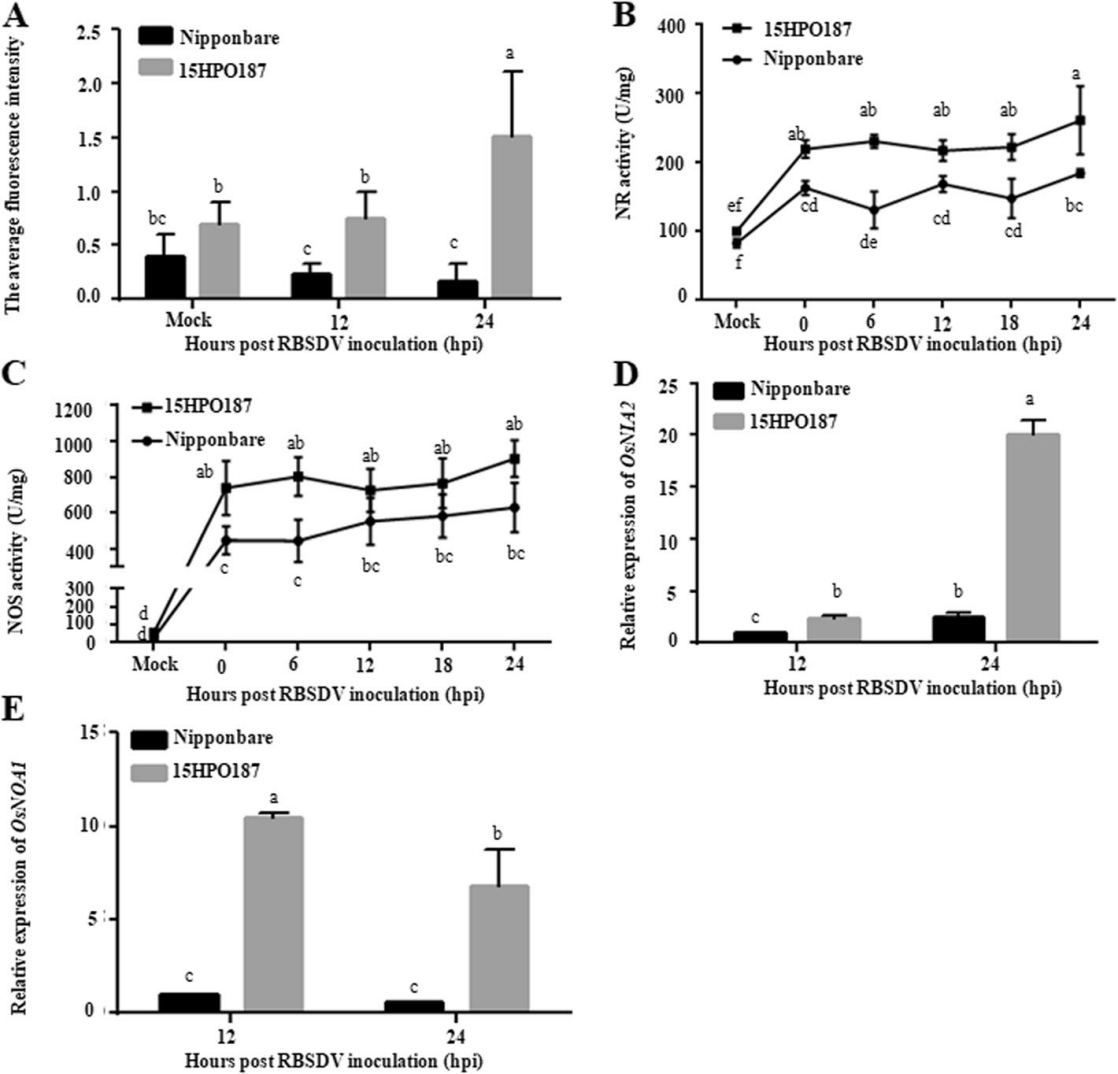
Function of endogenous NO in RBSDV infection. **a** Endogenous NO accumulation in the RBSDV-inoculated Nipponbare or 15HPO187 plants at various hpi. Ten plants were analyzed as ten biological replicates. The data represented the means ± SD of the ten replicates. Entire experiment repeated 3 times with similar results. **b** and **c** Activities of nitrate reductase (NR) and mammalian NO synthase (NOS)-like enzyme in the RBSDV-inoculated plants at various hpi. Ten plants were pooled together as one biological experiment. Images are representative of three independent biological experiments. The data represented the means ± SD of the three replicates. **d** and **e** Expressions of *OsNIA2* and *OsNOA1* in the RBSDV-inoculated plants at various hpi. Five plants were pooled together as one biological experiment. The data represented the means ± SD of the three biological replicates. Entire experiment repeated 3 times with similar results. Different low case letters above the bars indicate the statistical differences between the treatments using the Duncan’s multiple test

To compare the productions of endogenous NO in the RBSDV-infected Nipponbare and 15HPO187 plants, stem cross sections were collected from the inoculated plants at 12 and 24 hpi, and stained with DAF-FM DA as previously described (Xie et al. 2013; Xie et al. 2014) followed by Confocal Microscopy. Results of the experiments showed that the fluorescence signal representing endogenous NO production in the RBSDV-infected 15HPO187 stems was significantly stronger than that in the Nipponbare stems, while there was no significant different in endogenous NO production between uninfected Nipponbare and 15HPO187 plants (Fig. 2a). To support this finding, we analyzed the time course of NR and NOS activities in the RBSDV-inoculated 15HPO187 and Nipponbare plants. Agreed with the changes of NO production, the activities of NR and NOS were much higher in the RBSDV-infected 15HPO187 plants than that in the RBSDV-infected Nipponbare plants until 24 hpi, but the uninfected Nipponbare and 15HPO187 plants did not display such changes (Fig. 2b, c). qRT-PCR results showed that the relative expression level of *OsNIA2* was significantly higher in the RBSDV-inoculated 15HPO187 plants than that in the Nipponbare plants at 12 and 24 hpi (Fig. 2d). Similar result was also obtained for *OsNOA1* (Fig. 2e).

### NO Production Was Affected by the Treatment of Exogenous NO after RBSDV Infection

**Fig. 3:**
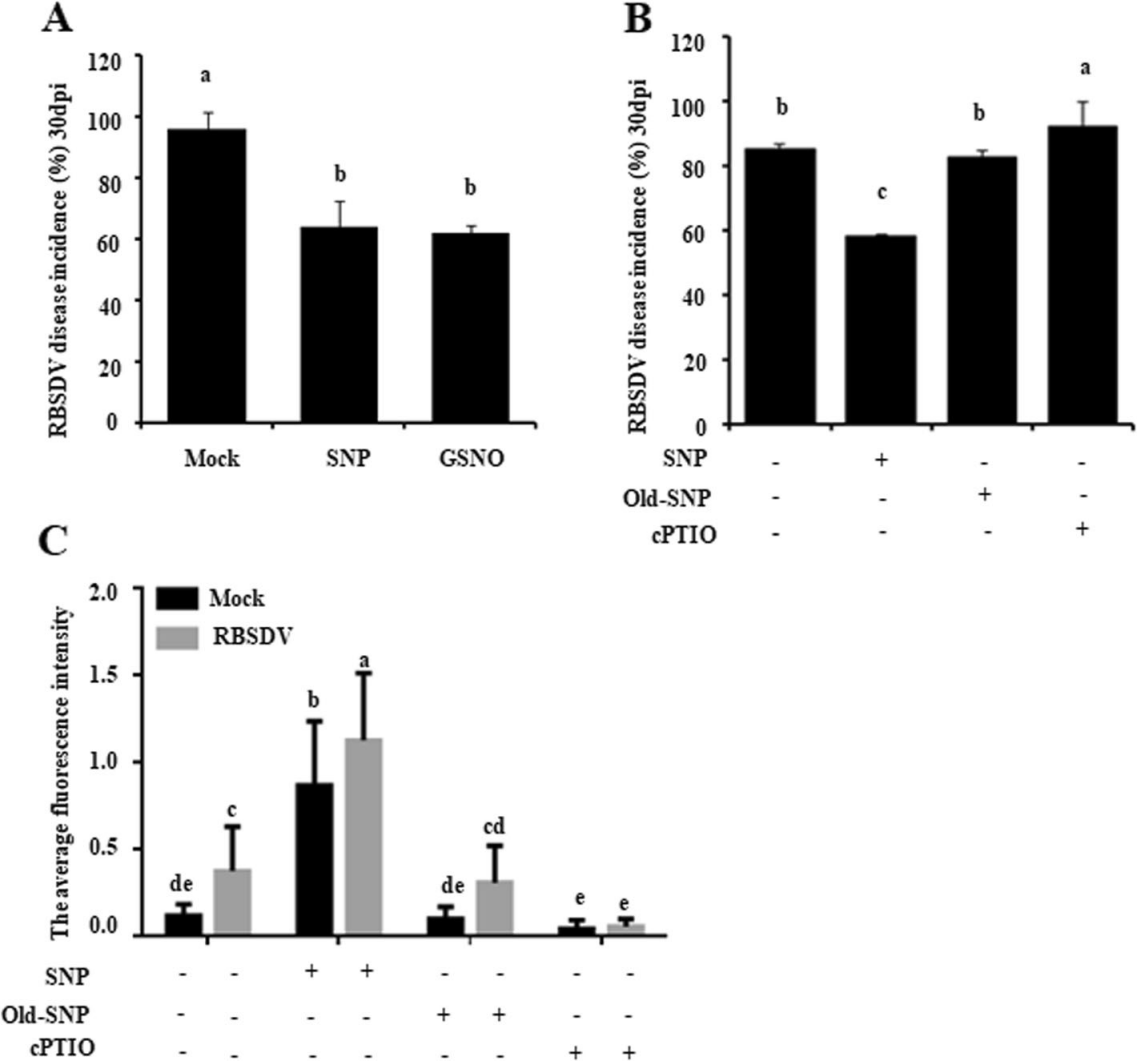
Changes in Nipponbare rice resistance to RBSDV infection in response to NO-releasing reagents or NO scavenger. **a** RBSDV disease incidence of the 50 μM SNP or GSNO pre-treated rice. RBSDV inoculation was carried out using RBSDV viruliferous or non-viruliferous SBPHs. Results were from three independent experiments with 30 plants per treatment. The data represented the means ± SD of the three replicates. **b** RBSDV disease incidence of the 50 μM SNP or old SNP, or 100 μM cPTIO pre-treated Nipponbare rice. Plants pre-treated with water only were used as controls. RBSDV disease incidences were determined at 30 dpi. Results were from three independent experiments with 30 plants per treatment. The data represented the means ± SD of the three replicates. **c** NO production in the stems of the SNP-, old SNP- or cPTIO pre-treated mock- or RBSDV-inoculated Nipponbare plants. Stem sections with about 3 mm thick, were stained with DAF-FM DA, and examined and imaged under a confocal laser scanning microscope. Then, the signal representing NO production in stems was captured and analyzed using the ZEN software. Ten plants were analyzed as ten biological replicates per treatment. The data represented the means ± SD of the ten replicates. Entire experiment repeated 3 times with similar results. Different low case letters above the bars indicate the statistical differences between the treatments, by using the Duncan’s multiple test

To further assess the role of endogenous NO in above process, Nipponbare seedlings were treated with the NO-releasing reagent SNP or GSNO followed by RBSDV inoculation. In the initial experiments, the seedlings were pre-treated with 10, 50, or 100 μM SNP, and the result showed that the pre-treatment of rice seedlings with SNP, especially at 50 μM SNP, could significantly reduce RBSDV disease incidence (Fig. S2). Consequently in the later experiments, the rice seedlings were pre-treated with 50 μM SNP or GSNO followed by RBSDV inoculation. By 30 dpi, RBSDV disease incidence of the SNP- or GSNO-treated plants were decreased significantly compared with the plants pre-treated with water (Mock) (Fig. 3a). As expected, the pre-treatment of rice seedlings with 50 μM old SNP, a negative control of SNP, did not change RBSDV disease incidence significantly (Fig. 3b). When rice seedlings were pre-treated with cPTIO, a NO specific scavenger, the incidence of RBSDV disease was significantly increased.

To demonstrate the specificities of SNP and cPTIO on NO production in rice, we analyzed NO production in the stems of the SNP- or cPTIO-treated RBSDV-inoculated Nipponbare rice through DAF-2DA staining and Confocal Microscopy. Results of the experiments showed that the SNP-treated RBSDV-inoculated rice plants accumulated about 2.5 fold more NO compared with that in the water-treated RBSDV-inoculated plants (Figs. 3c, S3). In the same experiment, the cPTIO-treated RBSDV-inoculated rice plants showed a significant reduction of NO content compared with that in the water-treated RBSDV-inoculated plants. As expected, the level of NO in the old SNP-treated RBSDV-inoculated rice plants was similar to that in the water-treated RBSDV-inoculated rice plants. Combined with corresponding phenotypes in RBSDV disease incidence (Fig. 3a, b), these results indicate again that NO is a key regulator of rice resistance to RBSDV infection.

### *OsICS1* and Stress-Responsive Gene Expression in Response to NO

**Fig. 4:**
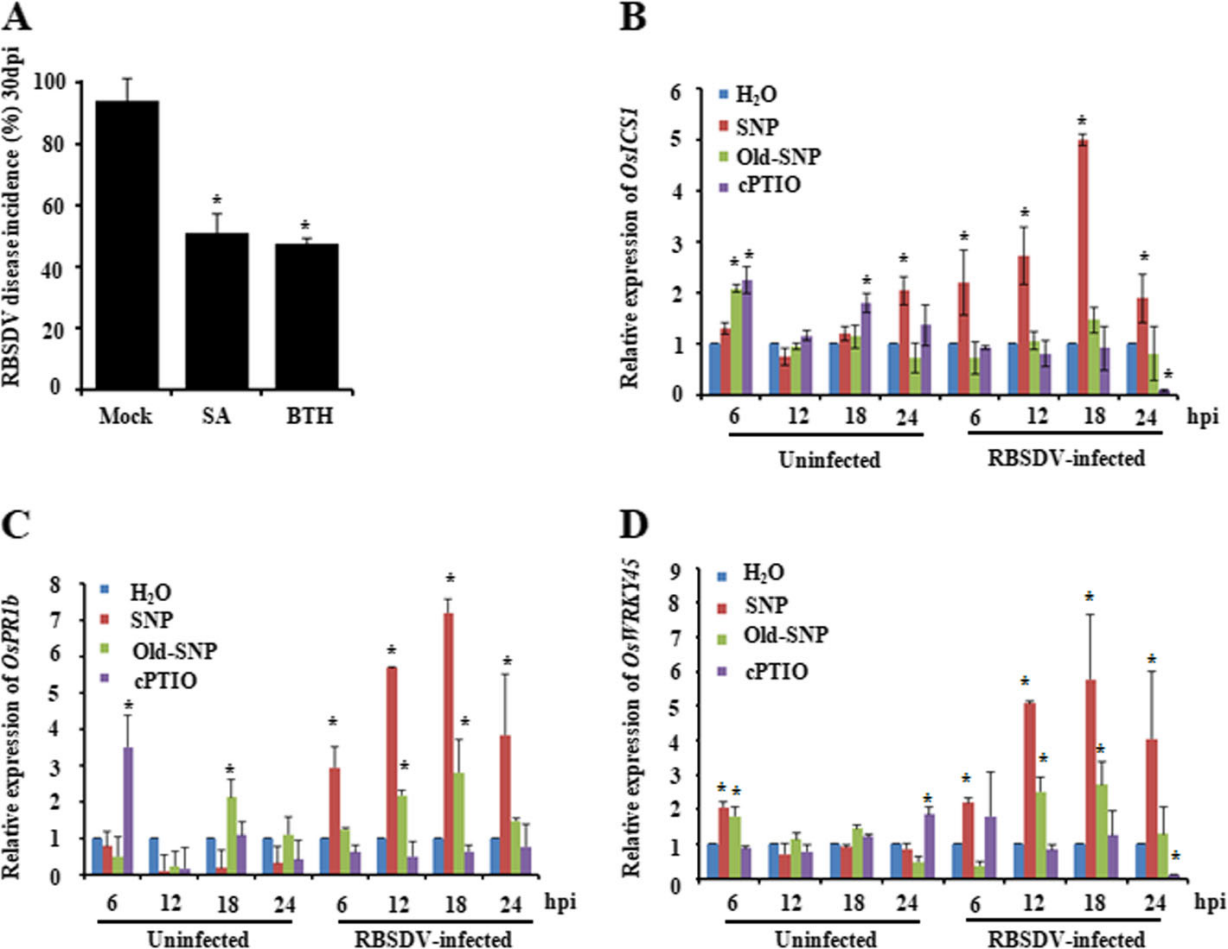
Changes in SA and defense responsive genes. **a** RBSDV disease incidence of the 500 μM SA- or 500 μM BTH pre-treated Nipponbare rice. 30 two-leaf stage rice seedlings of each cultivar were tests for disease incidence experiment. Images are representative of three independent biological experiments. The data represented the means ± SD of the three replicates. **b** to **d** Expressions of *OsICS1*, *OsPR1b* and *OsWRKY 45* in the SNP-, old SNP- or cPTIO-treated RBSDV-inoculated plants determined by qRT-PCR. Expressions of *OsUBC* and *OsActin1* in the rice plants were used as internal controls. The expression levels of *OsICS1*, *OsPR1b* and *OsWRKY 45* in the water-treated RBSDV-inoculated plants were set as 1.0. Five plants were pooled together as one biological experiment. The data represented the means ± SD of the three biological replicates. Entire experiment repeated 3 times with similar results. Statistical differences between treatments in each time point were determined by the Duncan’s multiple test, *p* < 0.05

Since salicylic acid (SA), indoleacetic acid (IAA), jasmonic acid (JA) and abscisic acid (ABA) are important regulators of plant growth and development, in this study, we further analyzed the levels of these phytohormones in the RBSDV-infected and non-infected rice plants at 24 h after RBSDV infection. The results showed that SA production was affected by RBSDV infection (Fig. S4). Meanwhile, the Nipponbare rice seedlings supplemented with 500 μM SA or BTH (a chemical analogues of SA) prior to RBSDV inoculation were also investigated. By 30 dpi, a significant reduction of RBSDV disease incidence was observed for the SA- or BTH-treated RBSDV-inoculated plants, compared with the water-treated RBSDV-inoculated plants (Fig. 4a). The result indicates that SA is also involved in the rice resistance to RBSDV infection.

To further investigate the relationship between NO and SA in rice, rice seedling plants were treated with SNP, old SNP or cPTIO followed by RBSDV inoculation. The expression levels of *OsICS1* (encoding isochorismate synthase, a major enzyme involved in SA biosynthesis), *OsPR1b* and *OsWRKY 45* in these treated plants were analyzed at 6, 12, 18, and 24 hpi, respectively. Results showed that the expression of *OsICS1* in the SNP-treated plants was significantly up-regulated by 6 hpi compared with that in the water-treated plants. This up-regulation was continued till 18 hpi followed by a decrease at 24 hpi. Although the up-regulation of *OsICS1* expression was significantly decreased at 24 hpi, it was still much higher than that in the water-treated plants (Fig. 4b). The expression of *OsICS1* in the cPTIO-treated plants was not reduced until 24 hpi compared with that in the water-treated plants. It was, however, much lower than that in the SNP-treated plants at 6 through 24 hpi. Meanwhile, the plants treated with old SNP showed no significant change of *OsICS1* expression compared with the water-treated plants. We also noticed that the expression of *OsICS1* in the old SNP-treated plants was much lower than that in the SNP-treated plants. The expression level of *OsICS1* was also detected under these treatments without RBSDV infection. The result showed that the changes of *OsICS1* in non-infected rice plants after various treatments were slight and lacked regularity (Fig. 4b).

The expressions of *OsPR1b* (6 through 24 hpi) and *OsWRKY 45* (12 through 24 hpi) were significantly up-regulated in the SNP-treated plants (Fig. 4c, d). On the other hand, the treatment of rice seedlings with cPTIO did not alter *OsPR1b* (except 12 hpi) or *OsWRKY 45* (except 24 hpi) expression compared with that in the water-treated plants. When compared with the SNP-treated plants, the cPTIO-treatment suppressed *OsPR1b* (6 through 24 hpi) or *OsWRKY 45* (12 through 24 hpi) expression. In the old SNP-treated plants, the expression of *OsPR1b* was induced at 12 and 18 hpi. These increases were, however, much lower than that in the SNP-treated plants. Similar tendencies were observed in the changes in *OsWRKY 45* transcripts when old SNP was applied. The expression levels of *OsPR1b* and *OsWRKY 45* were also detected under these treatments without RBSDV infection. The results showed that the changes of these two genes in non-infected rice plants after various treatments were slight and lacked regularity (Fig. 4c and d). These results indicate strongly that NO production in the RBSDV infected rice plants can regulate SA production and the stress-responsive genes.

### Profiles in Protein *S*-Nitrosylation

**Fig. 5:**
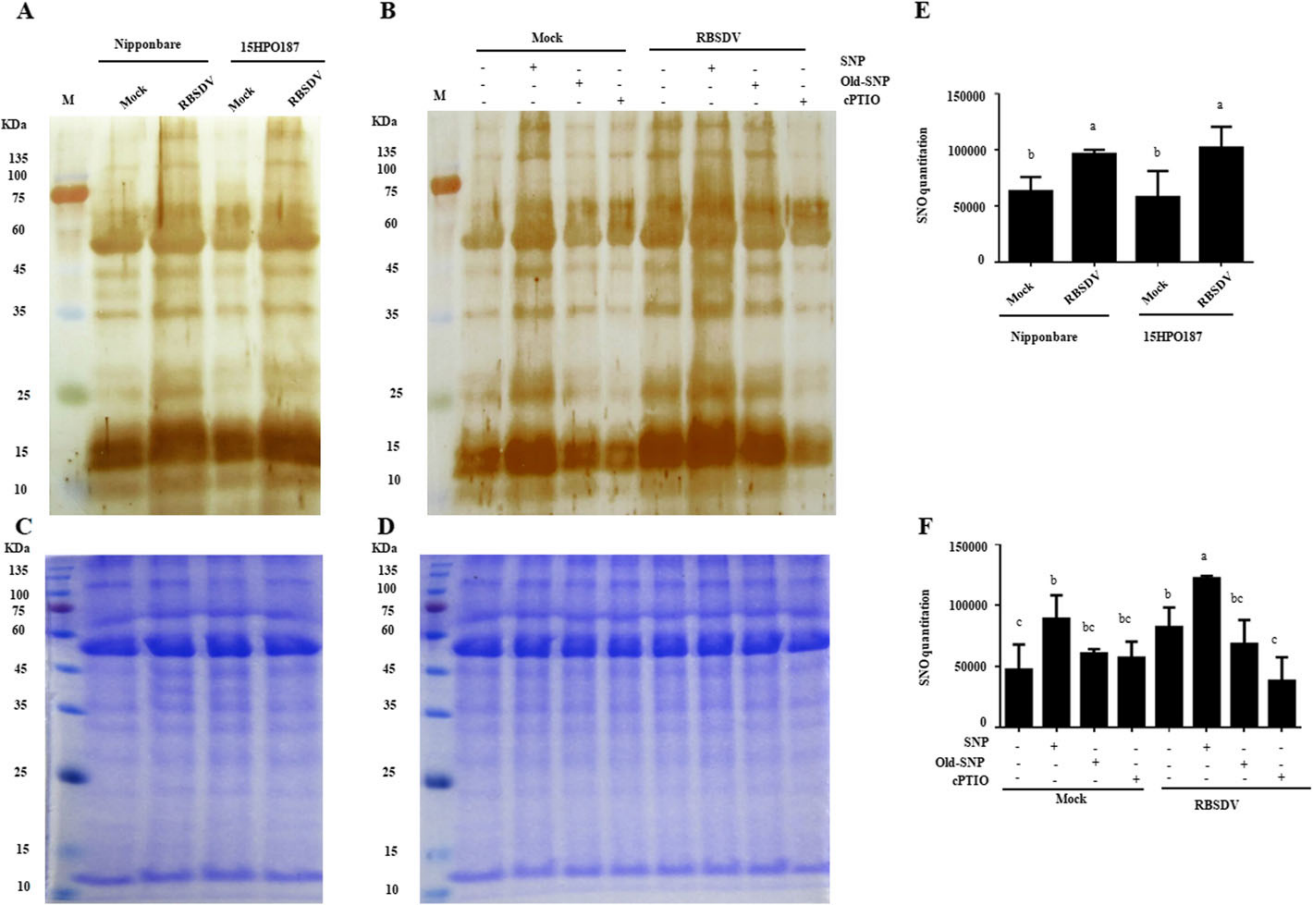
NO-dependent protein *S*-nitrosylation. **a** Total protein was isolated from the mock- or RBSDV-inoculated Nipponbare or 15HPO187 plants. The isolated protein samples were analyzed by a modified biotin switch method followed by western blot assay using a biotin specific antibody. Lanes with darker labeling signal indicate stronger protein *S*-nitrosylation. **b** Total protein was isolated from the SNP-, old SNP- or cPTIO-treated and mock- or RBSDV-inoculated plants and analyzed as described in (**a**). Numbers on the left slide of the gels are the size of protein markers. **c** and **d** Images of gels stained with Coomassie Brilliant Blue, and used to show equal protein loadings. Images are representative of experiments repeated at least three separate times. The blot was quantified using ImageJ software (http://rsbweb.nih.gov/ij/). The data represented the means ± SD of the three replicates. M, protein marker. All the experiment were repeated 3 times and representative results are shown

To investigate whether NO can mediate protein *S*-nitrosylation during RBSDV infection in rice, we analyzed RBSDV-inoculated Nipponbare and 15HPO187 plants, and compared them with the mock-inoculated plants using a modified biotin switch assay. Results showed that the level of protein *S*-nitrosylation was significantly increased in the RBSDV-inoculated Nipponbare and 15HPO187 plants (Fig. 5a and e). In this study, we also analyzed the SNP-, old SNP- or cPTIO-treated and RBSDV- or mock-inoculated Nipponbare plants. The results showed that the SNP treatment did intensify protein *S*-nitrosylation during RBSDV infection. When cPTIO or old SNP was individually used, the level of protein *S*-nitrosylation was not altered significantly, compared with that in the water-treated RBSDV-inoculated plants (Fig. 5b and f). This result indicates that NO produced during RBSDV infection in rice can *S*-nitrosylate proteins at post-translational level.

### Genetic Evidence Supported the Finding that NO Enhances Rice Resistance against RBSDV

**Fig. 6:**
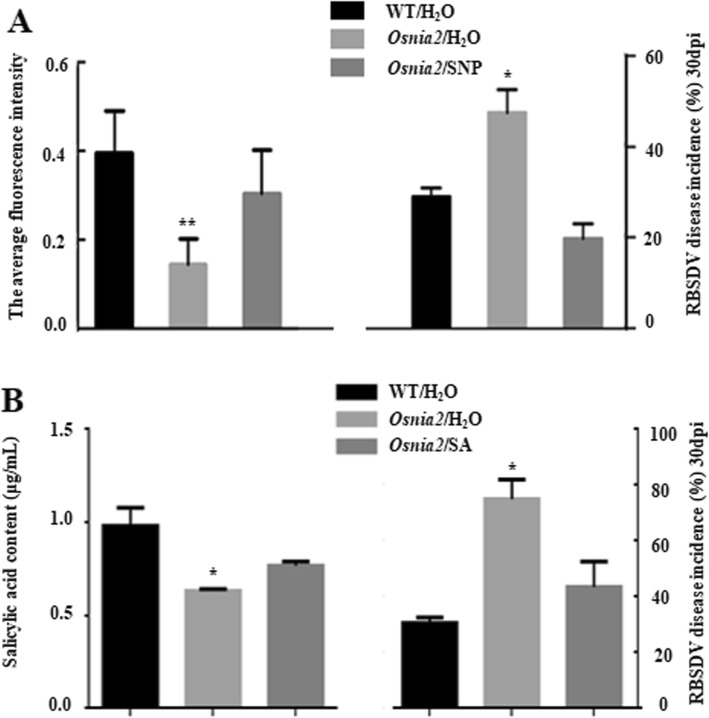
RBSDV disease incidence and NO production in rice cv. Dongjing (WT) and *Osnia2* mutant plants. **a** Left. NO production in the SNP-treated RBSDV-inoculated *Osnia2* mutant plants and RBSDV-inoculated Dongjing and *Osnia2* mutant. NO production analysis was assessed as described in Fig. 3. Ten plants were analyzed as ten biological replicates per treatment. The data represented the means ± SD of the ten replicates. Entire experiment repeated 3 times with similar results. Right. Dongjing and *Osnia2* mutant rice plants were pre-treated with SNP followed by RBSDV inoculation. Disease incidences of Dongjing and *Osnia2* mutant rice were determined from three independent experiments with 30 plants per treatment. The data represented the means ± SD of the three replicates. **b** Left. The amount of endogenous SA in the SA-treated RBSDV-inoculated *Osnia2* mutant plants and RBSDV-inoculated Dongjing and *Osnia2* mutant. The data represented the means ± SD of the three replicates. Entire experiment repeated 3 times with similar results. Right. Dongjing and *Osnia2* mutant rice plants were pre-treated with SA followed by RBSDV inoculation. Disease incidences of Dongjing and *Osnia2* mutant rice were determined from two independent experiments with 30 plants per treatment. The data represented the means ± SD of the two replicates. Statistical differences between the treatments were determined by the Duncan’s multiple test, *p* < 0.01 or *p* < 0.05

AtNIA1 and AtNIA2 were reported to control NO production in Arabidopsis, and play important roles in Arabidopsis resistance to pathogen invasions (Lozano-Juste and León 2009; Lozano-Juste and León 2010). In this study, we analyzed wild type (WT) rice cv. Dongjing and its *Osnia2* mutant plants for their responses to RBSDV infection. Results of confocal microscopy shown in Fig. 6a indicated that the level of NO in the infected *Osnia2* mutant plants (18 hpi) was much lower than that in the WT plants; while, when SNP was administrated, NO level was restored, approximately to the similar levels in the WT. By 30 dpi inoculation, the disease incidence of the mutant plants was significantly higher than that shown in the WT plants, which was decreased obviously when SNP was used. We also pretreated SA on *Osnia2*, and the results showed that the phenotype of pretreated mutant could be recovered to the similar level of WT. And the amount of endogenous SA was decreased in *Osnia2* mutant compared with the WT, while SA treatment could recover the endogenous SA accumulation, similar to that level in WT (Fig. 6b). These genetic findings thus support the above results, indicating that NO plays an important role in rice resistance to RBSDV infection through the SA pathway.

## Discussion

Although multiple RBSDV resistant or tolerant rice cultivars have been utilized in rice production, the mechanism(s) controlling this resistance or tolerance are still not fully elucidated. In our previous studies, we found several RBSDV resistant rice cultivars, including cv. 15HPO187. This finding promoted us to identify the key factor(s) regulating rice RBSDV resistance. In our test, the increase of NO level in the Nipponbare rice plants by RBSDV-infection supports the conclusion that NO production in plants can be induced upon pathogen invasion (Zou et al. 2018). Compared to the NO production in the Nipponbare plants (a sensitive cultivar) with that in the 15HPO187 plants (a resistant cultivar), we have found that RBSDV infection in the 15HPO187 plants produced much more NO than that in the Nipponbare plants (Figs. 1, 2a). Because NO is known to be catalyzed by nitrate reductase (NR) or mammalian NO synthase (NOS)-like enzyme (Besson-Bard et al. 2008; Simontacchi et al. 2015), we analyzed the activities of these two enzymatic activities in RBSDV-infected Nipponbare and 15HPO187 plants. Consistent with the NO results shown above, the activities of these two enzymes were significantly up-regulated in the infected plants, especially in the 15HPO187 plants (Fig. 2b, c). We speculate that the higher NOS and NR activities in the RBSDV infected 15HPO187 plants caused more NO production, and more NO in the plants caused a higher resistance to RBSDV infection.

Multiple NO production inducers and scavengers are now available for NO function studies. For example, sodium nitroprusside (SNP) is a commonly used NO-releasing compound or NO donor in many laboratories (Lindermayr et al. 2005; Zhao et al. 2018). 2-(4-Carboxyphenyl)-4, 4, 5, 5-tetramethylimidazoline-1-oxyl-3-oxide (cPTIO) is a commonly used NO specific scavenger in many studies (Liu et al. 2015; Deng et al. 2016). To validate the role of endogenous NO in rice resistance to RBSDV infection, we treated Nipponbare rice seedlings with two NO-releasing reagents SNP and GSNO, and then inoculated plants with RBSDV. Result of the initial experiment showed that rice plants pre-treated with 50 μM SNP showed much lower RBSDV disease incidence than the plants pre-treated with 10 or 100 μM SNP (Fig. S2). Consequently, in all later experiments, both NO-releasing reagents were diluted to 50 μM prior to use. As expected, the plants pre-treated with NO-releasing reagent showed a significant reduction of RBSDV disease incidence (Fig. 3a, b). To further confirm this finding, we pre-treated rice seedlings with a NO scavenger (cPTIO) followed by RBSDV inoculation. Result of this experiment showed that the NO production in the rice plants pre-treated with cPTIO was significantly suppressed, leading to a significant increase of RBSDV disease incidence in the plants (Fig. 3c, d). Therefore, we suggested that NO is an important regulator of rice resistance to RBSDV infection.

Song and Goodman (2001) reported that the size of TMV-induced local lesions in tobacco leaves was reduced after the NO induction, and the bioactivity of NO was dependent on the function of salicylic acid (SA) generated through the systemic acquired resistance (SAR) signaling pathway. Phytohormones were also known to regulate plant defense responses to biotic and/or abiotic stresses (Klessig et al. 2000; Freschi 2013; Ji et al. 2016; Qi et al. 2016). This leads us to investigate the interaction between NO and SA using SNP-, or cPTIO-treated rice plants. Results of the experiment showed that, similar to the response of NO, the production of SA in the SNP-treated RBSDV-inoculated rice plants was significantly induced while SA production in the cPTIO-treated RBSDV-inoculated plants was obviously suppressed (Fig. 4c). Based on these findings, we conclude that RBSDV infection in rice increases NO and SA production, and higher productions of NO and SA in the 15HPO187 plants might result in a resistance to RBSDV infection.

Previous studies have indicated that plant *PR1* gene is involved in the SA signaling (Klessig et al. 2000; Šašek et al. 2014). Transcription factor *OsWRKY45* was also reported to be responsive to SA or BTH during rice defense against rice blast and bacterial blight pathogen infections (Shimono et al. 2007; Shimono et al. 2012; Li et al. 2014). In addition, *OsICS1* was shown as a key gene for SA biosynthesis (Choi et al. 2015). To further validate SA induction, we treated rice seedlings with SNP or cPTIO prior to RBSDV inoculation and then analyzed the expressions of two SA responsive genes *OsPR1b* and *OsWRKY 45*, and *OsICS1* transcript. As expected, the SNP-treated RBSDV-inoculated plants showed up-regulated expressions of these three genes, especially at 18 hpi (Fig. 4d to f). In contrast, the cPTIO-treated RBSDV inoculated plants showed significantly reduced expressions of *OsICS1*, *OsPR1b* and *OsWRKY 45* compared with that in the water-treated RBSDV-inoculated plants. In addition, plants pre-treated with SA or BTH showed much lower RBSDV disease incidences than the water-treated RBSDV-inoculated control plants (Fig. 4b). Many research laboratories have used protein *S*-nitrosylation assay to investigate the functions of NO in plant responses to biotic and abiotic stresses (Jaffrey and Snyder 2001; Lindermayr et al. 2005; Yun et al. 2011). In this study, our protein *S*-nitrosylation assay showed that the induction of NO production (Fig. 2a) in the SNP-treated mock-inoculated or RBSDV-inoculated plants did increase protein *S*-nitrosylation (Fig. 5). As expected, the increase of protein *S*-nitrosylation was not observed in the cPTIO-treated mock-inoculated or RBSDV-inoculated plants, due mainly to the lack of NO production. Combined with the corresponding phenotypes (Fig. 3), our results strongly suggested that NO-targeted *S*-nitrosylation might be involved in rice resistance to RBSDV infection. Certainly, the direct target of *S*-nitrosylation during this process should be fully elucidated in the near future.

It was reported that Arabidopsis *Atnia1/2* mutant plants had lower NR activity than the WT plants (Wilkinson and Crawford 1993). To provide genetic evidence showing that rice plants with less endogenous NO were more susceptible to RBSDV infection, we challenged our *Osnia2* mutant rice seedlings with RBSDV, and compared them with the WT parental plants. Results presented in Fig. 6 showed that the *Osnia2* mutant plants had much lower NO content than that in the WT plants challenged with RBSDV. Consistently, the RBSDV disease incidence of the *Osnia2* mutant rice was much higher than that of the WT parental rice (Fig. 6). Above responses could be obviously abolished when exogenously applied SNP was used. This genetic result further supports our conclusion that NO has a key role in rice resistance to RBSDV infection.

**Fig. 7:**
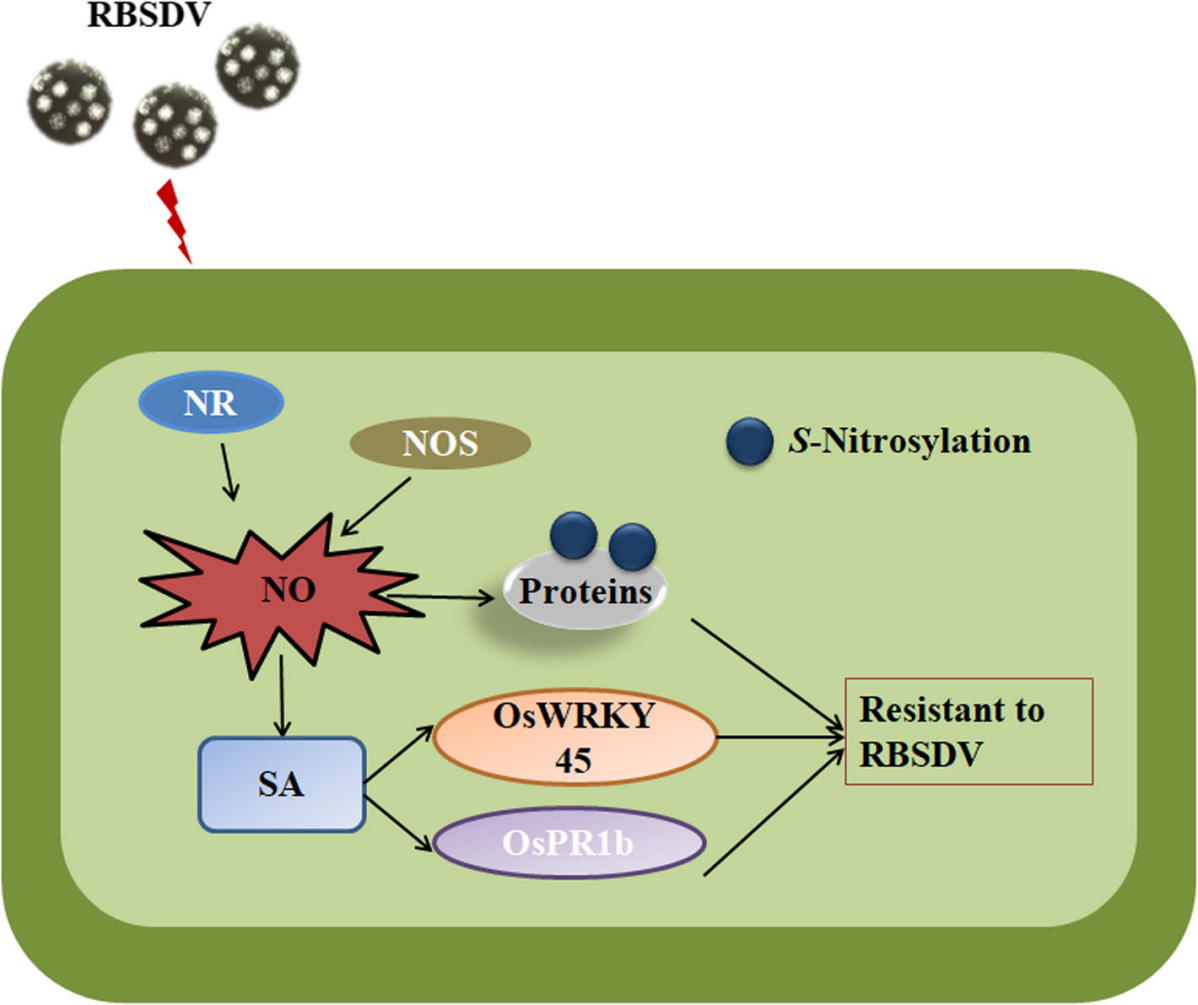
A working model for NO-dependent rice resistance to RBSDV infection. RBSDV infection in rice plants causes an early NO burst. The accumulated endogenous NO catalyzed by NR and NOS activities, acts as an inducer to trigger SA production. The accumulated SA up-regulates the expressions SA responsive host defense genes, thus leading to rice resistant to RBSDV

## Conclusions

Taken together, we propose a working model for the NO-dependent rice resistance to RBSDV infection (Fig. 7). RBSDV infection in rice causes an early NO burst. The accumulated NO might act as an inducer to induce SA production. It was reported that the SA-dependent signaling can be divided into two branches: the *OsNPR1* transcription factor dependent branch and the *OsWRKY45* transcription factor dependent branch, as reported previously (Sugano et al. 2010; Nakayama et al. 2013; Takatsuji 2014). NPR1 monomers are synthesized in cytoplasm and then translocated into nucleus to bind TGACG motif-binding factor (TGA1), a transcription factor controlling the expressions of many downstream defense genes, to form NPR1-TGA1 complexes to trigger the expressions of specific R genes as reported (Lindermayr et al. 2010). Previous genetic studies have shown that *OsWRKY45* is involved in the SA signaling pathway and is responsible for the activation of rice defense genes against *Magnaporthe oryzae* and *Xanthomonas oryzae* pv *oryzae* infection (Nakayama et al. 2013; Bakshi and Oelmüller 2014). In this study, SA induced the expressions of *OsWRKY45* and *OsPR1b,* which is involved in inhibition of virus infections. However, whether *OsWRKY45* and *OsPR1b* inhibit the virus infection is still unknown until a genetic analysis is taken. We consider that our study improves our current understanding on the interaction between rice and RBSDV, and possibly other rice-infecting viruses.

## Methods

### Chemicals

Unless otherwise specified, all chemicals used in this study were purchased from Sigma-Aldrich (Sigma-Aldrich, St Louis, MO, USA). In this study, SNP and GSNO were used as NO-releasing reagents, diluted to 50 μM in water, and applied to soil in pots (9 cm in diameter and 14 cm tall, 25 mL/pot) with rice seedlings (Parani et al., 2004). At 12 h post SNP or GSNO application, the rice seedlings were inoculated with RBSDV viruliferous small brown planthopper (SBPH). The old SNP solution was produced by placing the SNP solution inside test tubes and stored them under the light for more than 10 days to exhaust NO. The old SNP solution was obtained as a negative control by maintaining a 50 μM SNP solution for at least 2 d in the light in an open tube to eliminate NO as described (Tossi et al. 2009; Han et al. 2014). 2-(4-carboxyphenyl)-4, 4, 5, 5-tetramethylimidazoline-1-oxyl-3-oxide (cPTIO), a NO specific scavenger, was diluted to 100 μM in water prior to use (Parani et al., 2004; Kong et al., 2012). Salicylic acid (SA) and 2, 1, 3-benzothiadiazole (BTH) were purchased from MDBio (MDBio Inc., Taiwan, China), and diluted to 500 μM in water (SA was diluted by 1 mL ethanol first, then used water to constant volume), respectively. These reagents were applied individually to rice seedlings as described for SNP and GSNO above.

### Plant Growth and Virus Inoculation

For the experiments described in Figs. 1, 2, 3, 4 and 5, RBSDV viruliferous or non-viruliferous SBPH-inoculated rice seedlings cv. Nipponbare (a susceptible cultivar to RBSDV) and/or 15HPO187 (a resistance cultivar to RBSDV) were used. For the experiment described in Fig. 6, seedlings of rice cv. Dongjing and its *Osnia2* mutant were used (Sun et al. 2016).

SBPH nymphs were allowed to feed on the RBSDV-infected rice plants for 72 h as previously described (Zhou et al. 2010). The nymphs were transferred onto healthy rice seedlings cv. Wuyujing No. 3 and allowed to feed on them for another 10–12 days at 25 °C. The percentage of viruliferous SBPH was then determined by a dot enzyme-linked immunosorbent assay (Dot-ELISA) as described (Zhou et al., 2004). For virus inoculation, 30 two-leaf stage rice seedlings were randomly selected and each seedling was inoculated with three viruliferous SBPHs for 3 days as described (Zhou et al. 2011). Seedlings inoculated with non-viruliferous SBPHs were used as controls. Leaf tissues were sampled from the inoculated plants at various hours post SBPH inoculation (hpi), or various days post SBPH inoculation (dpi). The collected leaf tissues were immediately frozen in liquid nitrogen and stored at − 80 °C till use.

### Analysis of NOS or NR Activity

The NOS or NR activity was determined following the previous method (Zhao et al. 2009). For NOS activity, 200 μL of protein extract in the reaction mixture (100 mM PBS, pH 7.0, containing 1 mM L-arginine, 2 mM MgCl_2_, 0.3 mM CaCl_2_, 4 μM tetrahydrobiopterin, 1 μM flavin adenine dinucleotide, 1 μM flavin mononucleotide, 0.2 mM DL-dithiothreitol, 0.2 mM NADPH) was detected spectrophotometrically at 340 nm. For NR activity, the produced nitrite was determined spectrophotometrically at 540 nm by the addition of 1 ml of 1% (w/v) sulfanilamide in 3 M HCl together with 1 ml of 0.02% (v/v) N-(1-naphthyl)- ethylenediamine.

### Quantification of Endogenous NO in Rice Stems

The samples were collected at 18 h after RBSDV infection. Cross sections (3 mm thick) were cut from stems of viruliferous or non-viruliferous SBPH-inoculated rice plants, infiltrated with a NO fluorescent probe (10 μM 4-amino-5-methyl-amino-2′,7′-di-fluorofluorescein diacetate [DAF-FM DA]) diluted in a 20 mM Hepes-NaOH buffer, pH 7.2, followed by 15 min incubation in the dark (Balcerczyk et al. 2005; Qi et al. 2017).

After thorough rinse in the Hepes-NaOH buffer, the sections were examined, imaged, and processed using a Zeiss LSM 710 confocal laser scanning microscope equipped with a ZEN software (Carl Zeiss, Oberkochen, Germany). The excitation wavelength was set at 488 nm and the emission wavelength was set at 500–530 nm. More than 10 rice plants were analyzed for each treatment.

### Quantification of Endogenous SA in Tissue Samples

The assay was determined following the previous method (Novák and Floková 2018). Rice leaf samples (500 mg each) were collected from the assayed plants at 24 hpi, ground individually in liquid nitrogen, and then homogenized in 10 mL extraction buffer of isopropanol/H2O/hydrochloric acid (200:100:0.2). The crude leaf extracts were shaked for 12 h followed by adding 15 mL dichloromethane. The organic phase evaporated in vacuo to dryness, which was dissolved in 400 μl 50% methanol. The samples were filter with 0.22 μm organic filter membrane, then were taken for analysis by HPLC-MS (1260 FLD Serial No.DEAB001256).

### Total RNA Isolation and Quantitative Reverse Transcription Polymerase Chain Reaction (qRT-PCR)

Total RNA was isolated from rice leaf samples (100 mg tissue per sample) using Trizol reagent (Invitrogen, Gaithersburg, MD, USA). Concentration of total RNA in each sample was determined using a NanoDrop 2000 Spectrophotometer (Thermo Fisher Scientific, Wilmington, DE, USA). cDNA was synthesized using one μg total RNA per 20 μL reaction using the PrimeScript™ RT reagent Kit with a gDNA Eraser (Takara, Dalian, China). qPCR was then performed using the SsoFast EvaGreen® Supermix (Bio-Rad) on a Bio-Rad iQ5 qRT-PCR system. The expression levels of *OsUBC* and *OsActin1* were determined and used as internal controls as previously reported (Fang et al. 2015; Lu et al. 2016). qPCR primers specific for RBSDV *P10*, *OsNOA1*, *OsNIA2*, *OsPR1b*, *OsWRKY45* or *OsICS1* are listed in the Supplementary Table 1. The qRT-PCR results were calculated using the 2^-ΔΔCt^ method reported previously (Livak and Schmittgen 2001).

### Immunoblot Assay of *S*-Nitrosylated Proteins

Assays of *S*-nitrosylated proteins were done as described previously (Jaffrey and Snyder 2001; Forrester et al. 2009; Qi et al. 2017). Total protein was isolated from the collected tissues at 24hpi and the *S*-nitrosylated biotin-labeled proteins were separated in 12% SDS-PAGE gels under the non-reducing condition. The protein bands were blotted onto polyvinylidene difluoride (PVDF) membranes followed by protein detection using an anti-biotin antibody (Abcam antibodies, Cambridge, UK). The blot was quantified using ImageJ software (http://rsbweb.nih.gov/ij/). Protein loadings were estimated through Coomassie Brilliant Blue staining.

### Statistical Analysis

All the experiments conducted in this study were done in triplicate. Results of the experiments were presented as the means of the three independent experiments ± standard deviation (SD). Statistical analyses were performed using the SPSS 18.0 software (Armonk, USA) and the Duncan’s multiple tests. Differences at *p* < 0.05 were considered to be significant.

## Supplementary information

**Additional file 1: Fig. S1.** The phenotype of RBSDV-inoculated Nipponbare and 15HPO187. Representative three plants of each cultivar were shown. Scale bars = 5 cm.

**Additional file 2: Fig. S2**. Disease incidence of RBSDV-inoculated Nipponbare pre-treated with different concentrations of SNP. Nipponbare plants were pre-treated with different concentrations of SNP for 12 h followed by RBSDV inoculation using viruliferous SBPHs. 30 two-leaf stage rice seedlings of each cultivar were tests for disease incidence experiment. Images are representative of three independent biological experiments. The data represented the means ± SD of the three replicates.

**Additional file 3: Fig. S3**. NO production in the stems by different treatments in mock- (left column) or RBSDV-inoculated (right column) Nipponbare plants. NO production in the stems of the SNP-, old SNP- or cPTIO pre-treated mock- (left column) or RBSDV-inoculated (right column) Nipponbare plants. Stem sections were about 3 mm thick, stained with DAF-FM DA, and examined and imaged under a confocal laser scanning microscope. Up and right corner inserts are bright filed images of the stem sections. Scale bars = 200 μM. Images are representative of biological replicates from experiments repeated at least three times.

**Additional file 4: Fig. S4**. Concentrations of phytohormones in RBSDV-infected (RBSDV) or Non-infected (Mock) Nipponbare plants**.** Accumulations of three different phytohormones in the RBSDV-infected or non-infected rice plants were determined by high efficiency liquid chromatography method (ACQUITY UPLC Xevo TQ, Waters, USA). 100 mg plant tissues were used as one biological experiment. Images are representative of three independent biological experiments. The data represented the means ± SD of the three replicates.

**Additional file 5: Table S1.** Primers used for quantitative reverse transcription polymerase chain reaction (qRT-PCR).

## Data Availability

All data supporting the conclusions of this article are provided within the article (and its Additional files).

## Abbreviations

RBSDV: *Rice black-streaked dwarf virus*
NO: Nitric oxide
SNP: Sodium nitroprusside
GSNO: Nitrosoglutathione
cPTIO: 2-(4-carboxyphenyl)-4, 4, 5, 5-tetramethylimidazoline-1-oxyl-3-oxide
NOS: NO synthase;
NR: Nitrate reductase
SA: Salicylic acid
BTH: 2, 1, 3-benzothiadiazole
