# Supplementary figures and images for: Nitric Oxide Enhances Rice Resistance to Rice Black-Streaked Dwarf Virus Infection
Source: Rice (N Y). 2020 Apr 14;13:24. doi: 10.1186/s12284-020-00382-8 (PMC7156532; doi:10.1186/s12284-020-00382-8)

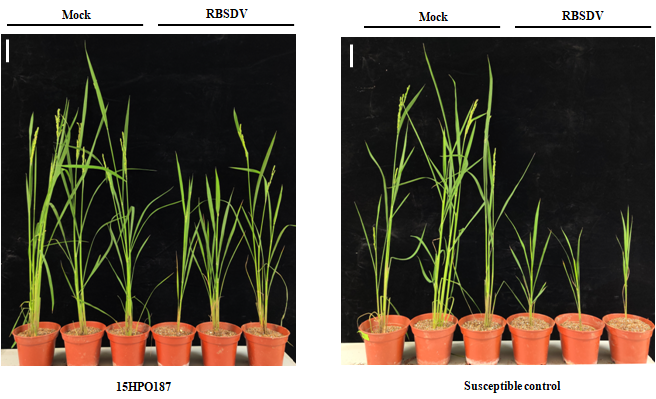

Supplement: Supplementary file 1 — Additional file 1: Fig. S1. The phenotype of RBSDV-inoculated Nipponbare and 15HPO187. Representative three plants of each cultivar were shown. Scale bars = 5 cm. [file 12284_2020_382_MOESM1_ESM.tif]

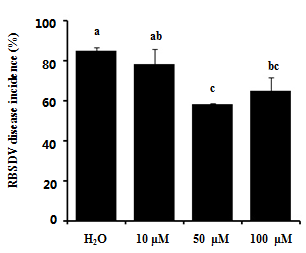

Supplement: Supplementary file 2 — Additional file 2: Fig. S2. Disease incidence of RBSDV-inoculated Nipponbare pre-treated with different concentrations of SNP. Nipponbare plants were pre-treated with different concentrations of SNP for 12 h followed by RBSDV inoculation using viruliferous SBPHs. 30 two-leaf stage rice seedlings of each cultivar were tests for disease incidence experiment. Images are representative of three independent biological experiments. The data represented the means ± SD of the three replicates. [file 12284_2020_382_MOESM2_ESM.tif]

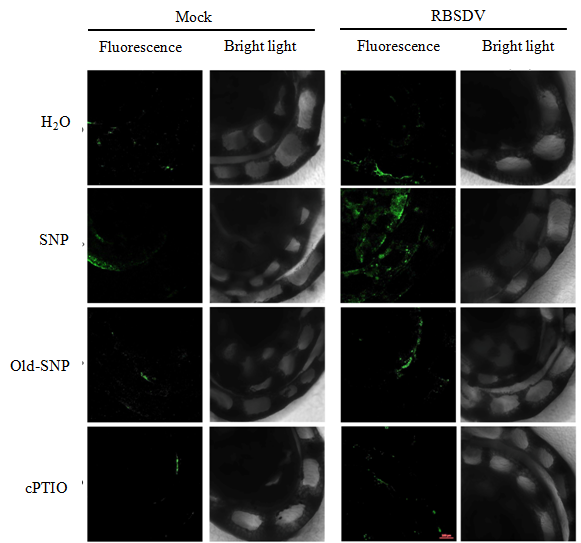

Supplement: Supplementary file 3 — Additional file 3: Fig. S3. NO production in the stems by different treatments in mock- (left column) or RBSDV-inoculated (right column) Nipponbare plants. NO production in the stems of the SNP-, old SNP- or cPTIO pre-treated mock- (left column) or RBSDV-inoculated (right column) Nipponbare plants. Stem sections were about 3 mm thick, stained with DAF-FM DA, and examined and imaged under a confocal laser scanning microscope. Up and right corner inserts are bright filed images of the stem sections. Scale bars = 200 μM. Images are representative of biological replicates from experiments repeated at least three times. [file 12284_2020_382_MOESM3_ESM.tif]

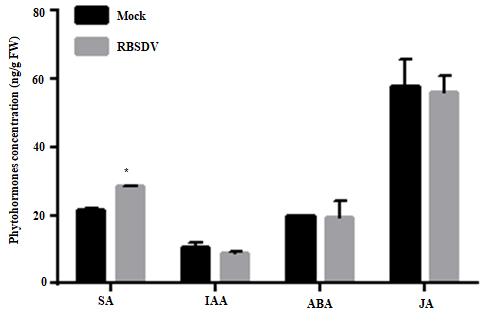

Supplement: Supplementary file 4 — Additional file 4: Fig. S4. Concentrations of phytohormones in RBSDV-infected (RBSDV) or Non-infected (Mock) Nipponbare plants. Accumulations of three different phytohormones in the RBSDV-infected or non-infected rice plants were determined by high efficiency liquid chromatography method (ACQUITY UPLC Xevo TQ, Waters, USA). 100 mg plant tissues were used as one biological experiment. Images are representative of three independent biological experiments. The data represented the means ± SD of the three replicates. [file 12284_2020_382_MOESM4_ESM.tif]
