# Supplementary material for: Nitric Oxide Enhances Rice Resistance to Rice Black-Streaked Dwarf Virus Infection
Source: Rice (N Y). 2020 Apr 14;13:24. doi: 10.1186/s12284-020-00382-8 (PMC7156532; doi:10.1186/s12284-020-00382-8)
Supplement: Supplementary file 5 — Additional file 5: Table S1. Primers used for quantitative reverse transcription polymerase chain reaction (qRT-PCR). [file 12284_2020_382_MOESM5_ESM.pdf]

Supplementary Table S1. Primers used for quantitative reverse transcription polymerase chain reaction (qRT-PCR)

Gene name	Accession number	Primer sequence (5′→3′)
OsUBC	AK059694	F-CCGTTTGTAGAGCCATAATTGCA
		R-AGGTTGCCTGAGTCACAGTTAAGTG
OsActin1	AK100267	F-CTCCCCCATGCTATCCTTCG
		R-TGAATGAGTAACCACGCTCCG
RBSDV *P10*	AF227205	F-GCCCCACGTTGCATCTTC
		R-TGTTGGGCAAAGTGCTAGTTTC
OsNOA	Os02g0104700	F-TGCTTCTGTGGTTGGGAC
		R-TCTAAGGGCACGGTGTTT
OsNIA2	AK102363	F-ACTGGTGCTGGTGCTTCTGG
		R-CGGCTGGGTGTTGAGGGACT
OsPR1b	AK107926	F-ACGCCTTCACGGTCCATAC
		R-AAACAGAAAGAAACAGAGGGAGTAC
OsWRKY45	EF143611	F-TCAGTGGAGAAGCGGGTGGTG
		R-GGGTGGTTGTGCTCGAAGGAG
OsICS1	LOC9268489	F-TATGGTGCTATCCGCTTCGAT
		R-CGAGAACCGAGCTCTCTTCAA
